# Electrophoretic µPAD for Purification and Analysis of DNA Samples

**DOI:** 10.3390/bios12020062

**Published:** 2022-01-24

**Authors:** Natascha Katharina Heinsohn, Robert Raimund Niedl, Alexander Anielski, Fred Lisdat, Carsten Beta

**Affiliations:** 1 InventicsDx GmbH, Magnusstrasse 11, 12489 Berlin, Germany; r.niedl@inventicsdx.com (R.R.N.); a.anielski@inventicsdx.com (A.A.); 2 Biological Physics, Institute of Physics and Astronomy, University of Potsdam, Karl-Liebknecht-Strasse 24/25, 14476 Potsdam, Germany; 3 Biosystems Technology, Institute of Life Sciences and Biomedical Technologies, Technical University of Applied Sciences, Hochschulring 1, 15745 Wildau, Germany

**Keywords:** microfluidic paper analytic device (µPAD), patterning glass microfiber, fiber-electrophoresis chip, DNA, imprinted electrodes, cross layer chip, polymerase chain reaction (PCR), purification

## Abstract

In this work, the fabrication and characterization of a simple, inexpensive, and effective microfluidic paper analytic device (µPAD) for monitoring DNA samples is reported. The glass microfiber-based chip has been fabricated by a new wax-based transfer-printing technique and an electrode printing process. It is capable of moving DNA effectively in a time-dependent fashion. The nucleic acid sample is not damaged by this process and is accumulated in front of the anode, but not directly on the electrode. Thus, further DNA processing is feasible. The system allows the DNA to be purified by separating it from other components in sample mixtures such as proteins. Furthermore, it is demonstrated that DNA can be moved through several layers of the glass fiber material. This proof of concept will provide the basis for the development of rapid test systems, e.g., for the detection of pathogens in water samples.

## 1. Introduction

The development of analytical methods and devices for the identification and characterization of biological samples by DNA detection is of broad significance in biotechnology and medical applications. The integration of distinct processes into a miniaturized format is particularly advantageous when it comes to aspects such as portability, speed of measurement and analysis, disposability, decreased waste production, and low manufacturing costs [1,2,3,4]. Electrophoresis based on the migration of particles under the influence of an electric field represents a significant method in the analysis of biomolecules. Nowadays, complex protein and DNA mixtures are mostly separated by size in combination with charge using gel-based electrophoresis methods (e.g., agarose or polyacrylamide gels).

The beneficial properties of paper-based materials such as high surface areas, accessible hydroxyl groups, and biocompatibility make them an interesting candidate for microfluidic applications. It is a low-cost material, easily available, and biodegradable. Thus, paper can be used as a supporting matrix for the separation of different substances [5,6,7]. Applications of fiber materials in µPADs also exploit further advantages such as fluid flow through capillary forces, dry storage, or covalent coupling of chemicals and biomolecules on the fiber [8,9,10]. Additionally, combinations with other materials have been demonstrated, e.g., with thermoresponsive hydrogels for an on-chip separation of analytes [11]. Moreover, diverse 3D structures can be built by folding in several layers [12]. In addition, paper materials have become popular in the field of sensors as they allow cost-effective production and measurements at the point of need. Several detection methods including colorimetric, chemiluminescent, fluorescent, and electrochemical assays have been adopted to paper-based analytical devices [13,14,15,16,17,18,19,20,21,22].

Fiber-electrophoresis, also known as ionography, was established in 1948 for the investigation of different kinds of electrically charged, ionized substances or uncharged substances that are converted into charged complexes in solution [23]. A strip of wetted cellulose filter paper served as a bridge between two buffer reservoirs and charged particles migrated under the influence of an electric field toward the counter electrode. The technology has been predominantly used for the separation and identification of proteins, amino acids, as well as polyamines or complex ions [24,25,26]. For the detection, the papers were dried and sprayed with a solution of ninhydrin, butanol, or bromocresol-green to observe bands of the migrated samples. In order to separate purine, pyrimidine, and nucleotides, a paper electrophoresis system for nucleic acid derivates was described [27]. For the separation of serine, aspartic acid, and lysine on a cellulose-based device, an electro-generated chemiluminescence under strongly alkaline conditions at 330 V was developed [28]. A low-voltage paper-based origami was developed for the detection of fluorescent molecules and proteins in a patterned cellulose fiber containing a buffer reservoir with introduced Ag/AgCl electrodes [29]. The electrophoretic separation of synthetic RNA mimicking viral and human RNA was published recently [30]. The investigation of the basic electrophoretic migration of fluorescent dyes was carried out in experiments using isotachophoresis (ITP) [31]. Sample ions concentrate into zones in order of their mobility using a discontinuous buffer system. Cellulose-based origami with consistent fiber alignment was also used for the migration of artificial DNA by ITP [32].

Glass fibers have been used for the electrophoretic separation of organic substances, including polysaccharides, and for differentiating certain specimens of glycogen from various sources [33,34]. Glass microfiber material is preferred over cellulose paper because complexing between polysaccharides and the fiber material is avoided [35]. Microfibers made of borosilicate glass are heat-stable and show a high loading capacity and rapid flow rate. For our purpose, we have chosen glass microfiber in order to avoid complexing between DNA and the fiber material and to obtain better background signals due to the low autofluorescence of glass microfiber compared to cellulose papers during fluorescence measurement. Previously, long strips of fiber (7 × 40 cm) were connected between two buffer tanks and used at high voltages (120 V for 10 h) to distinguish energy storing polysaccharides, for example, from calf liver, seaweed, or birch wood [34]. In order to create defined reaction areas and fluid transportation pathways on the paper, different patterning and structuring methods have been applied such as wax or inkjet printing [8,36,37], photolithography [38], etching [39], laser cutting [40], or plasma treatment [41].

In this work, we describe a new glass fiber-based electrophoresis system with imprinted electrodes and demonstrate the fast and simple separation of DNA, not only from artificial but also complex biological sample mixtures such as genomic bacterial DNA. The separation can be combined with detection via an intercalating dye and offers the opportunity for further processing due to the easy recovery from the fiber by elution, which is appropriate for integration into µPADs. For the preparation of the glass microfiber-based electrophoresis chips, we developed a transfer printing process, which also allows implementation of different geometries of the fiber material. Additionally, this glass microfiber-electrophoresis can be carried out with simple and cost-efficient equipment.

## 2. Materials and Methods

### 2.1. Reagents and Equipment

The ColorQube 8570 printer and solid ink (wax) 108R00931 was obtained from Xerox (Norwalk, CT, USA), the overhead film from Office Tree (Las Vegas, NV, USA), and the transfer press 173,777 from Helo GmbH (Rastdorf, Germany). The PCB Printer Voltera V-One and all accessories for dispensing the electric paste were purchased from Voltera (Kitchener, ON, Canada). The carbon paste C2000802P2 was acquired by Sun Chemical (Parsippany, NJ, USA), gold-coated contact fingers by Würth (Künzelsau-Gaisbach, Germany), and the crocodile clamps and Voltacraft VC820 Multimeter digital by Conrad Electronics (Hirschau, Germany). The construction of the board and the circuit diagram was part of this work; all other electronic components, e.g., board, resistors, and cable, were obtained from Reichelt Elektronik (Sande, Germany). GelRed was obtained from Biotium (Fremont, CA, USA) and the 1 kb DNA ladder, lyophilized from Carl Roth (Karlsruhe, Germany). The Olympus DSX 500 optical microscope and the 5× objective MPLFLN5XBDP were obtained from Olympus (Tokyo, Japan). The Digital Microscope was obtained from Andonstar (Shenzhen, China). The glass microfiber filters Whatman GF/F, the peqGOLD 1 kb DNA ladder, the Chemi Premium Imager, agarose, gel chamber, power source PerfectBlueL, One-Step Blue protein stain, and all other consumables (Petri dish, pipette tips, etc.) were obtained from VWR (Darmstadt, Germany). *Legionella pneumophila Philadelphia* was obtained from DSMZ (Braunschweig, Germany). Genomic DNA was extracted using an alkaline extraction buffer containing 125 mM NaOH, 1 mM EDTA, 0.1% Tween 20, and a neutralizing buffer containing 125 mM HCl and 10 mM Tris HCl optimized from Brewster and Paoli [42]. The protocol was adapted to room temperature and the incubation time was reduced to 5 min.

### 2.2. Polymerase Chain Reactions

Primer sequences were selected from an alignment of nucleotide sequences of the 16S rRNA gene of *Legionella pneumophila Philadelphia* from NCBI (Reference NR_074231.1). The bacteria were cultured on BCYE agar plates from Xebios (Düsseldorf, Germany), containing antibiotics for selectivity, at 37 °C for three days. Genomic DNA was extracted using the QIA-quick Polymerase chain reaction (PCR) purification Kit (Qiagen, Hilden, Germany).

PCR was performed in a 10 µL reaction mixture consisting of 1 µL of 10× Taq reaction buffer (Biozym Scientific, Oldendorf, Germany), 2 µL of deoxyribonucleoside triphosphate (dNTPs) (8 mM, Thermo Fisher Scientific, Waltham, MA, USA), 0.4 µL of Primer forward 5′-GGTAATACGGAGGGTGCGAG-3′ and reverse 5′-ACTTCTGGTGCAACCCACTC-3′ (10 µM, Sigma Aldrich, St. Louis, MO, USA), 0.1 µL of Taq-Polymerase (Biozym Scientific, Oldendorf, Germany), and 0.5 µL of genomic template DNA. The PCR thermal profile consisted of an initial incubation of 1 min at 95 °C, followed by 33 cycles of 15 s at 95 °C, 15 s at 55 °C, and 15 s at 72 °C. Amplification was performed with the peqSTAR cycler (VWR, Darmstadt, Germany). Agarose gel electrophoresis was carried out in the 1 × TAE buffer at 120 V for 75 min. Detection after gel electrophoresis was accomplished with the Gel documentation system Chemi Premium Imager (VWR, Darmstadt, Germany).

Real-time PCR was performed in a 20 µL reaction mixture consisting of 10 µL of 2× InnuMix qPCR SyGreen (Analytik Jena, Jena, Germany), 1 µL of Primer forward 5′-GGTAATACGGAGGGTGCGAG-3′ and reverse 5′-ACTTCTGGTGCAACCCACTC-3′ (10 µM, Sigma Aldrich, St. Louis, MO, USA), and 0.5 µL of genomic template DNA. The PCR thermal profile consisted of an initial incubation of 2 min at 95 °C, followed by 40 cycles of 20 s at 95 °C, and 60 s at 56 °C. Amplification, detection, and data analysis were performed with the Real-Time PCR Thermal Cycler qTower^3^ (Analytik Jena, Jena, Germany).

### 2.3. DNA/Protein-Mix Sample Preparation

For the preparation of an artificial sample, 100 ng/µL of horseradish peroxidase (HRP) from Alfa Aesar (Ward Hill, MA, USA) was mixed with 120 ng/µL of a DNA fragment amplified by PCR with a size of 910 bp in phosphate buffer pH 7.0. The enzyme was detected via tetramethylbenzidine (TMB) substrate conversion by pipetting 40 µL of SeramunBlue fast, purchased from Seramun Diagnostics (Heidesee, Germany), onto the chip surface.

### 2.4. Preparation of the Electrophoresis Chip

**Figure 1:**
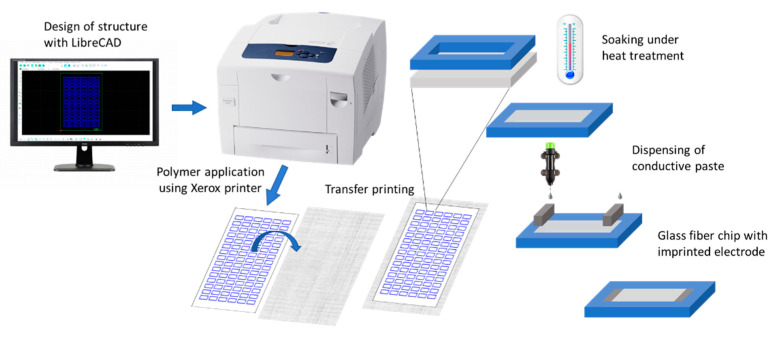
Schematic illustration of the transfer printing process of the glass microfiber. The pattern was generated via LibreCAD and printed on overhead film via a Xerox wax printer. Induced by heat application, the wax was transferred to the underlying fiber material. As electrodes within the fiber channel, a conductive carbon paste was applied using a dispenser.

The fabrication process is illustrated in Figure 1 For the design of the chip structure, LibreCAD as a free open-source CAD application software was used. A simple rectangular structure was chosen, multiplied on an A4 sheet, and finally exported as a pdf file. The solid ink of the Xerox Printer, commonly referred to as wax, is usually made of a mixture of resin-based thermoplastic polymers and mixed with dyes. For manufacturer’s details, please refer to Section 2.1. An overhead film can be inserted as with a conventional printer and the design was printed as a wax structure. After a heat treatment of the printed overhead film laying inverted on the glass microfiber filter at 160 °C for 30 s, the wax melted, penetrated the fiber interspaces, and resulted in a defined microchannel structure with an inner size of 9 × 1.9 cm. Here, a transfer press was used for heating to provide a flat and uniform barrier on the fiber material. The press (see Figure S1) containing a heating plate presses the overhead film and fiber together during the heating process and ensures close contact during transfer. A microscopic image section of the geometry before and after transfer is shown in Figure S2.

**Figure 2:**
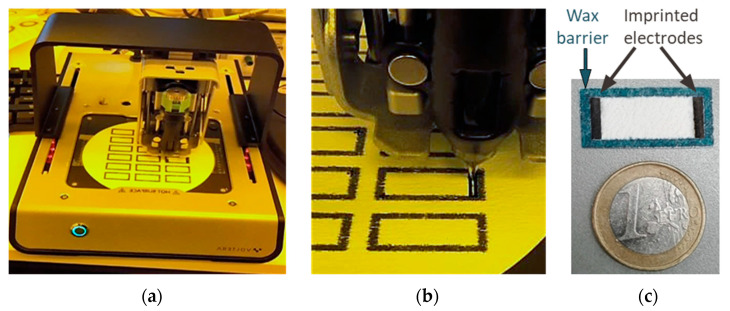
Images of the electrode manufacturing process. (**a**) Carbon paste applied to the glass microfiber using the Voltera printer. (**b**) Nozzle dispensing the carbon paste in a rectangular movement from the outside to the inside of the electrode area. (**c**) An individual electrophoresis sensor next to a euro coin.

The two ends within the microchannel of the fiber-based chip were treated with the conductive carbon paste, to create electrode structures that penetrate the fiber surface. A Voltera printer, usually used for circuit board prototyping, was used for the dispensing of the carbon paste onto the fiber chip and resulted in two rectangular electrodes, 2 × 10 mm, at each end of the chip. To prepare for printing of the electrodes, a cartridge was filled with a volume of 5 mL of carbon paste, avoiding air bubbles being included. After calibration for defining a fiber height profile for each glass microfiber filter sheet and alignment of the designed structure, the print height and pressure were adjusted. The dispensing parameters for the carbon paste on glass microfiber were 0.33–0.36 mm as the print height and 10–20 µm for the ink pressure. For fixation, the printed fibers were dried in an oven at 80 °C for 5 min. Pictures of the experimental setup used for the fabrication of the imprinted carbon paste electrode using the Voltera dispenser are shown in Figure 2. From one sheet of roundfilter Ø 14.2 cm, 18 fiber-electrophoresis chips can be fabricated within 15 min.

### 2.5. Description of the Measurement Setup

**Figure 3:**
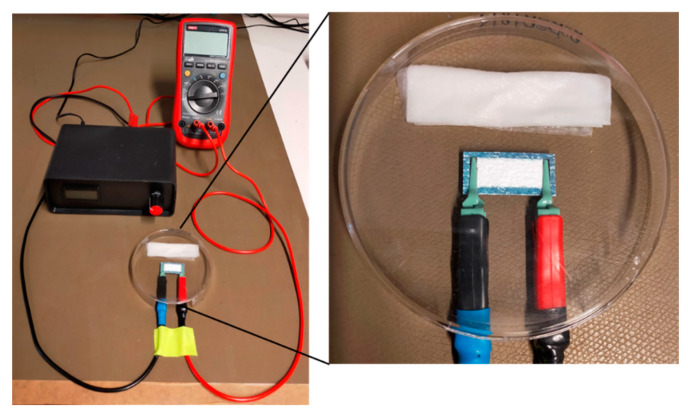
Photographs of fiber-electrophoresis experimental setup 1. The fiber-electrophoresis chip was placed between two crocodile clamps, next to a wetted wipe and covered by a petri dish to avoid evaporation. The clamps were connected to a stabilized voltage supply and a multimeter was integrated for current tracking.

To induce DNA movement, voltage was applied between the two printed electrodes. Alligator clamps were used for connecting the imprinted electrodes to a voltage source. A picture of the experimental setup is shown in Figure 3. A multimeter was integrated to detect the current during electrophoresis. During the project, the measurement setup was designed to be closer to the product, as measurements should be able to run in standardized devices at a later date, and it is referred to as measurement setup 2 in the following (see Figure S3).

### 2.6. DNA Electrophoresis on the Fiber Chip

After prewetting of the microchannel with 5 mM Tris buffer pH 8 as a running buffer, 1 µL of sample was pipetted in front of the negatively charge electrode (cathode) and a voltage of 45 V was applied for 15 min. After stopping DNA movement by switching off the voltage, die chips were cut into 4 pieces. Each of these areas were treated with 25 µL of elution buffer and incubated for at least 1 h. Afterward, the supernatants were loaded on a 1% agarose gel for gel electrophoresis to visualize DNA fragments. In this standard application, the DNA molecules are separated based on their size in the polysaccharide matrix with a vertically applied electric field, in which the small fragments run faster than the large ones. A 1 kb ladder is usually used as a size reference in gel electrophoresis but was chosen in this context as a defined DNA-sample consisting of several fragments with different sizes. For visualization of the movement of a 910 bp DNA fragment, a PCR product amplified from the 16S rRNA gene of *Legionella pneumophila Philadelphia*, fluorescence detection was used. In the separation section of the fiber chip, a fluorescence spot occurred after incubating the fiber upon electrophoresis with a 3 × GelRed staining solution diluted in 0.1 M NaCl for 3 min and imaging under UV-light. For testing the integrity of the DNA after field-driven movement on the glass fiber chip, an amount of 100 ng of the 910 bp fragment was used as template DNA for PCR and real-time PCR.

## 3. Results

### 3.1. Manufacturing of the Fiber-Based Electrophoresis Chip

**Figure 4:**
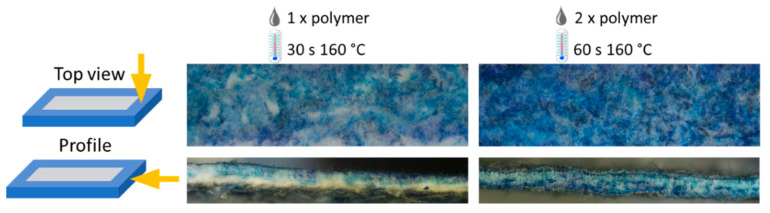
Penetration depth of wax soaked into glass microfiber. Top view and profile of a channel barrier are shown with a single and double amount of wax and 30 or 60 s of heat application to the glass microfiber.

For DNA movement and separation, a rectangular chip design was chosen with a separation length of 2.3 cm. The fluid-containing part was defined by wax printing. The designed structure was printed with a solid ink Xerox printer on an acetate sheet first and then transferred to the fiber material, as shown in Figure 1. Thus, the fiber can be prevented from being destroyed in the printer feed, which is essential because of the soft texture of the glass microfiber in comparison to commonly used cellulose fiber. The fiber area enclosed by the wax barrier remained hydrophilic. The penetration homogeneity and depth can be influenced by the amount of wax transferred and the time of heat application. Figure 4 shows that with twice the amount of blue colored wax and double the heating time, more of the white fiber areas penetrated. This also makes it possible to generate semi-sealed channel structures, e.g., if the speed of the capillary liquid motion should be influenced. Verification of the hydrophobic barrier was accomplished by buffer addition (Video S1).

After the area for fluid movement was defined by wax printing, two electrodes were fabricated on both ends of the fiber-based chip (see Figure 2). As the electrode material, carbon was chosen due to its low costs and easy processability. Carbon is also a preferred electrode material for electrochemical detection due to properties such as minimal fouling, a low background noise, and a large potential window for detection of organic molecules [43,44,45], although the latter aspects were not the focus of our investigations here. The carbon paste could directly be printed onto the glass fiber material (for details, see Section 2.4), resulting in a separation section of 1.9 cm in length.

### 3.2. Characterization of the Fiber-Based Electrophoresis Chip

The prepared electrodes were first characterized by resistance measurements. Although some variations could be found in the range from 500 to 2000 Ω/cm with medium values of around 1000 Ω/cm, no significant influence on the DNA separation was found. In order to analyze the glass fiber electrode interface and also to rationalize the different resistance values, microscopic pictures were taken for several electrodes on the fiber material (example shown in Figure S4). Some variations in the height of the carbon in the range from 220 µm to 500 µm could also be estimated here. This clearly shows that there was some variation in the deposition of the electrode material onto the glass fiber. However, one can see that the carbon material partly penetrated the fiber material, resulting in a 3D electrode within the glass fiber chip. The penetration depth can be estimated to be in the range from 50 to 150 µm. As the surface inside the fiber material is mainly responsible for the field formation, the structural variations found in the preparation play only a minor role and a homogeneous field for the migration of the DNA samples can be provided.

### 3.3. Conductive Fiber-Electrophoresis Chip for Movement of Nucleic Acid Mixtures

**Figure 5:**
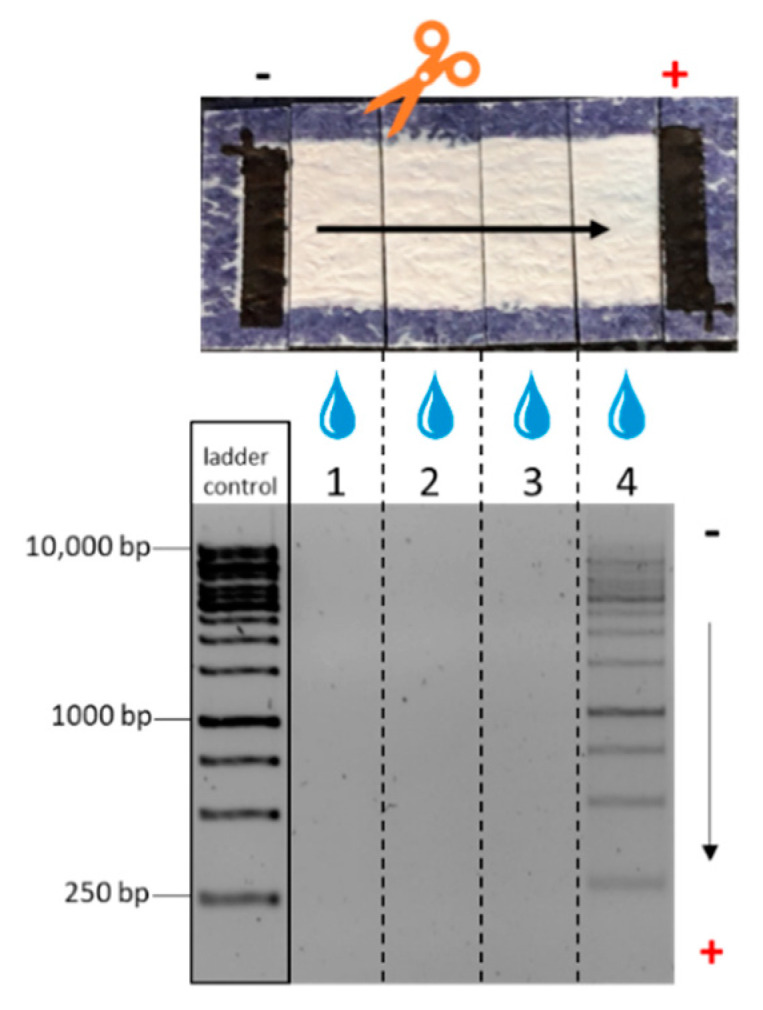
Fiber-based electrophoresis chip connected via imprinted electrodes and crocodile clamps to a voltage source. Upon the application of an electric field of 45 V, the sample migrated to the opposite electrode, verified by dividing and eluting of the chip area and visualized by gel electrophoresis. Each column on the agarose gel represents the eluted and loaded solution of one quarter of the chip area. As a control, the 1 kb ladder was loaded directly on the gel representing the initial sample composition.

The biochip is a simple two-electrode arrangement, in which the sample is moved by applying an electrical field between the electrodes. Here, a high-voltage approach was chosen and the DNA was applied in a small volume of 1 µL near the cathode (see also Section 2.6). After inducing DNA movement by electric field application, the chip area between the electrodes was cut into four equal parts, and the DNA was eluted and analyzed by standard gel electrophoresis. This approach was used as the sample was a mixture of DNA strands of different lengths from 250 to 10,000 bp. Figure 5 shows the result of such an experiment. The clear signal of DNA eluted from the fourth segment and the absence of DNA in the first, second, and third segments clearly proves the successful movement of the nucleic acids within the fiber material. Furthermore, it is obvious that all fragments moved independent of their size through the whole chip. As the average pore size of the material was relatively large (700 nm), no size discrimination of the DNA fragments over the small chip distance can be expected. The strong electric field applied will additionally change the structure of the DNA molecule, resulting in a longitudinal movement [42,43,44]. Thus, a molecular sieve effect could not be observed. Sample migration over the entire chip area, using lower electric fields compared to previous reported work (20 V/cm), was observed consistently within 5 min, offering a simple, fast, and robust method for DNA movement. Double-stranded RNA samples in cellulose-based electrophoresis or DNA in paper-based ITP experiments were performed under higher electric fields ranging from 160 to 210 V/cm for 6 to 10 min [30,32] or lower electric field strengths but substantially longer connection times, as shown for the separation of polysaccharides on the glass microfiber with 3 V/cm for 40 h [34].

**Figure 6:**
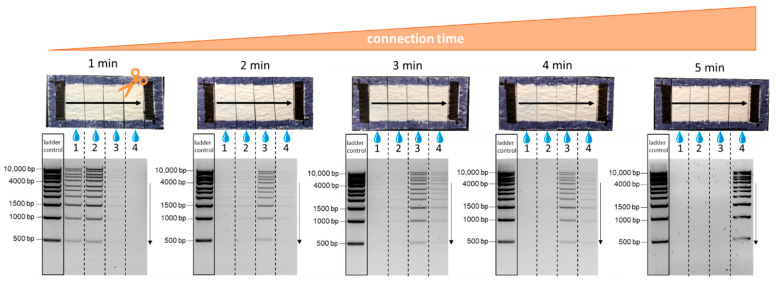
Fiber-based electrophoresis chips with printed electrodes after different connection times to the voltage source using measurement setup 2. A 1 kb ladder was used as a sample to investigate the migration of the DNA fragments of different sizes. From left to right, the individual chips were used for electrophoresis at 45 V for 1, 2, 3, 4, and 5 min.

In order to determine the migration rate of DNA fragments in the 1 kb ladder, fiber-electrophoresis chips were exposed to the electric field for different time durations. The connection time was varied from 1 to 5 min. In Figure 6, the different segments of the fiber chip are shown after elution and agarose gel electrophoresis. After 1 min, the sample spread over half of the chip area and started concentrating toward the anode after a field exposure of 2 to 3 min. After 5 min of voltage exposure, the DNA sample concentrated at the end of the fiber-electrophoresis chip. A size-dependent migration was again not detectable, because every fragment could be seen in every elution fraction. Based on these results, an average migration speed of about 4 mm/min could be estimated for the DNA samples. Even after increasing the connection time, the DNA sample did not run underneath the anode. Consequently, there was no risk of destroying the nucleic acid, allowing downstream detection reactions.

### 3.4. Movement of a Single DNA Fragment on the Electrophoresis Chip

In order to obtain more insight into the fast and well-defined movement of DNA strands within the 3D matrix of the glass fiber material, a single DNA fragment was investigated. *Legionella* as a waterborne pathogen is capable of colonizing anthropogenic water systems, infecting humans by inhalation of contaminated aerosols, and can cause severe pneumonia or even death. Consequently, the interest in innovative solutions for the purification and detection of this pathogen is very high. With this in mind, *Legionella* was used as the biological sample material for the work underlying the following sections of this paper. In the first step, a PCR product of a 910 bp DNA fragment amplified from the gene of *Legionella pneumophila Philadelphia* 16S rRNA was used as a sample for the fiber-based electrophoresis.

**Figure 7:**
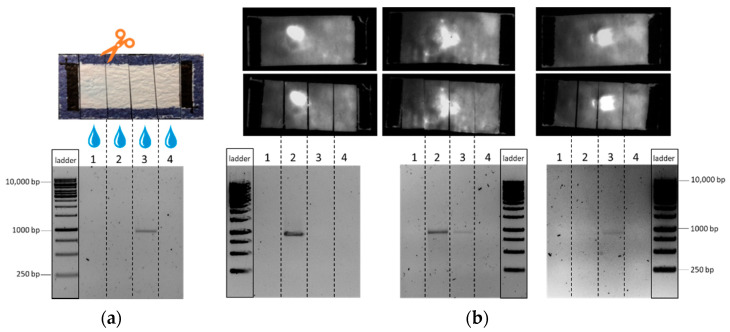
Images of a 910 bp DNA fragment amplified from *Legionella pneumophila Philadelphia* as a sample in fiber-based electrophoresis with measurement setup 2 and alternative visualization using post-staining with intercalating dye GelRed. (**a**) The fragment visualized on an 1% agarose gel after elution from the chip area. (**b**) Alternative visualization of the DNA fragment by staining with intercalating dye GelRed and no interference with subsequent elution.

To visualize DNA movement without destroying the fiber structure, an intercalating dye was used. In this way, the position of the single DNA fragment could be easily seen at different positions within the separation distance by the appearance of a fluorescent spot. This is illustrated in Figure 7. In order to verify that these observed spots were indeed connected to the presence of DNA, subsequent nucleic acid elution and agarose gel electrophoresis were used, identical to the experiments described before. As can be seen from Figure 7, the detection of the fluorescent spot corresponds well with the DNA detection by gel electrophoresis and supports the conclusions on the field-driven DNA movement within the glass fiber chip. The arrival of the sample at the end of the chip can also be visualized with the DNA stain, as shown in Figure S5.

**Figure 8:**
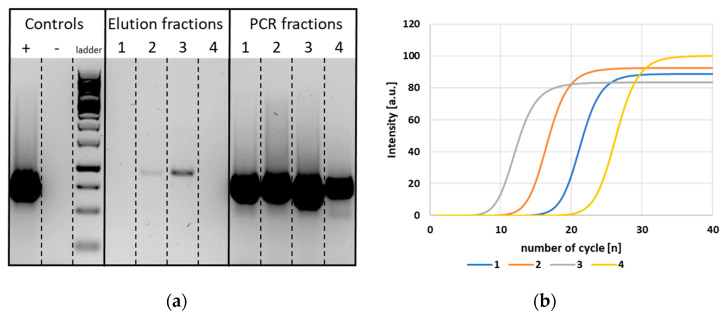
DNA samples eluted after fiber-electrophoresis and used as a template for PCR and real-time PCR. (**a**) A volume of 10 µL of each elution fraction was loaded directly on a 1% agarose gel. A PCR with primers for the 910 bp fragment and 1 µL of each elution fraction was performed and the final DNA samples were separated via agarose gel electrophoresis next to the elution fractions. (**b**) The graphs depict the intensities measured upon real-time PCR using the same primers and SyGreen. The numbers 1–4 refer to the position of the chip segments from left to right. The curves appearing first in real-time PCR were consistent with the most intense bands of the elution fractions. In both PCR and real-time PCR, the sample fragment loaded on fiber-electrophoresis can also be amplified if it is not seen in the elution fractions.

Another aspect of the DNA movement is connected to the properties of the transported nucleic acid. It is important to verify that the DNA samples can be used for downstream analysis, e.g., PCR after fiber-electrophoresis. This was tested with DNA eluted from the paper after the movement through the chip. A standard PCR with the primers for the 910 bp fragment and the elution fractions used as a template was set-up and the amplified DNA was loaded next to the pure elution fractions on the agarose gel. This is illustrated in Figure 8a and Figure S6a.

It can be clearly seen that much higher signals were registered after PCR amplification for all samples taken from different segments of the chip. Even for the segments where no DNA was detected by gel electrophoresis directly after the field-driven movement, PCR amplification could successfully multiply the residual target DNA. This means that some DNA remained in the fiber material, but this concentration was very small. During fiber electrophoresis, the DNA molecules were pulled to the positively charged electrode, but traces of the sample remained attached to the fibers. As PCR is a very sensitive method, these traces could be detected after amplification. However, the results also gave strong hints that the integrity of the DNA, which moved through the glass fiber network, can be retained.

As the quantification of DNA is difficult in agarose gel electrophoresis, real-time PCR was additionally performed with the same elution fractions. As can be seen from Figure 8b and Figure S6b, the signals showed a sigmoidal curve pattern. In the first cycles, the curves started at zero because no fluorescence signal could be detected by the instrument, due to the low amount of DNA. After an increase in the DNA product concentration amplification, a fluorescence intensity above the background level was reached and detected. The exponential amplification of the target sequence was then represented as a linear increase in fluorescence. A plateau was finally reached by the consumption of the reaction components (dNTPs). The higher the initial concentration of the target sequence, the fewer temperature cycles were required until the fluorescence increased. The signals obtained from real-time PCR in this work correspond very well to the results from gel electrophoresis of the pure elution fraction. The fluorescence signals occurring first in real-time PCR corresponded to the bands seen in gel electrophoresis of the pure elution fractions (elution fractions 3 and 2). Moreover, amplification signals of the remaining elution fractions could also be detected by real-time PCR but occurred later compared to the ones already seen in the elution fractions without amplification. This also implies that the small amounts of DNA remaining at the zone of sample loading or already reaching the counter electrode could not be detected by elution, but were not destroyed by the high electric field. In comparison, the negative control without template DNA showed no amplification signal (see Figure S7).

### 3.5. Separation of DNA from Other Potential Sample Components

**Figure 9:**
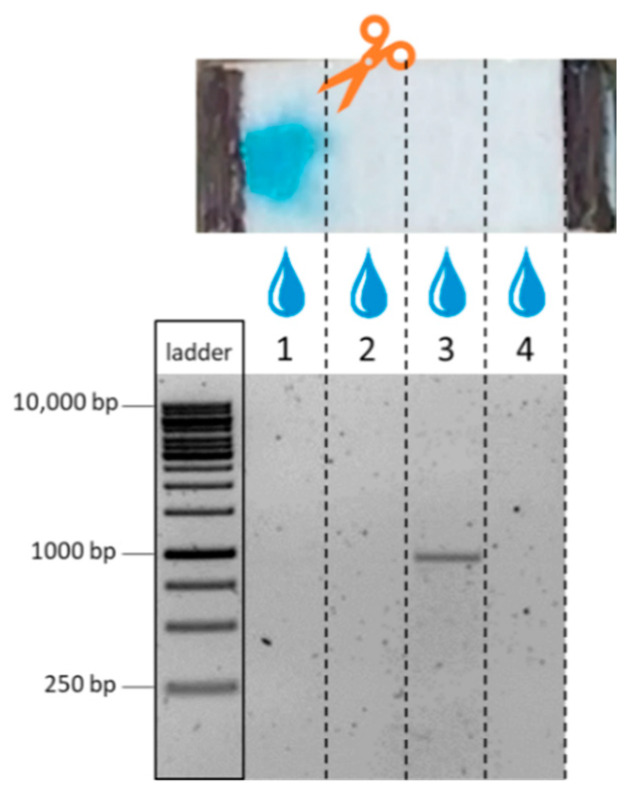
Separation of DNA fragment (910 bp) from HRP as representative of a potential protein contamination. Detection of the HRP after fiber-electrophoresis with crocodile clamps by TMB/H_2_O_2_ substrate oxidation to a blue color at the sample loading zone and detection of migrated DNA by elution and subsequent agarose gel electrophoresis from the third quadrant of the chip area.

In order to verify that the electric field-driven movement of DNA can also be used for an effective purification of a given sample, test experiments with more complex solutions were performed. First, the separation from proteins was studied. A mixture of the 910 bp DNA-fragment from *Legionella pneumophila Philadelphia* and horseradish peroxidase (HRP) as a medium-sized protein (44 kDa) was used. In order to visualize the position of the protein on the chip, the color reaction was performed with hydrogen peroxide and TMB. The substrate turnover of the enzyme indicated the localization of the enzyme after electrophoresis and verified the enzyme functionality after voltage exposure (see Figure 9 and Video S2). The blue color clearly indicates that the protein did not move in the experiment; the DNA fragment migrated as intended toward the anode, verified by the elution from the third chip fraction, see Figure 9. A combination of HRP detection by TMB and DNA staining via GelRed on the same chip followed by elution of the DNA sample was also possible (see Figure S8).

**Figure 10:**
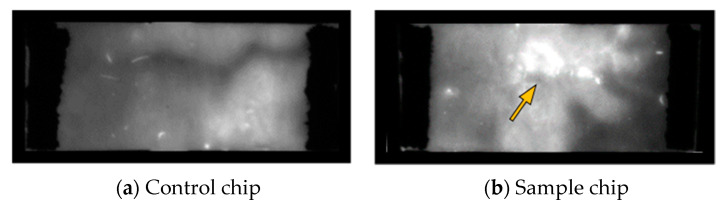
Purification of genomic DNA from cell lysate. (**a**) Staining of a control chip without DNA but lysis buffer as a sample with GelRed showing no fluorescent spots. (**b**) Fluorescent spots indicating the genomic DNA after fiber-electrophoresis with measurement setup 2 of a cell lysate from *Legionella pneumophila Philadelphia* stained with GelRed.

In the second step, the chip was investigated with respect to DNA purification from a cell lysate. For this purpose, a lysate of *Legionella pneumophila Philadelphia* (see Section 2.1) was loaded onto the glass fiber chip and an electric field was applied. Staining with GelRed after the fiber-electrophoresis visualized the DNA after migration, which can be seen in Figure 10b, while lysate containing proteins remained at the sample loading zone (see Figure S9). Thus, it is possible to specifically separate the genomic DNA from a complex biological mixture such as a bacterial cell lysate and to achieve a robust purification of the DNA molecules within a short time using this simple experimental setup.

### 3.6. Cross-Layer Chip Arrangement

**Figure 11:**
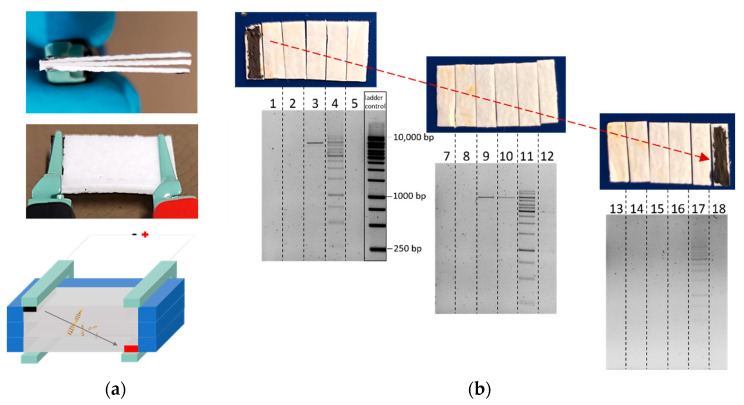
Cross-layer chip consisting of three layers of glass microfiber connected via the upper left and lower right electrode to generate an oblique migration direction through all layers. (**a**) Pictures and illustration of the cross-layer chip connected by crocodile clamps. The schematic illustration does not correspond to the actual height–length ratio. (**b**) Elution fractions of each layer after agarose gel electrophoresis.

In the last step of these developments, it was tested whether this simple setup can be used for a more complex DNA transport through several layers of the glass fiber material. Three glass fiber layers were stacked on top of each other, as shown in Figure 11, with the left electrode (cathode) remaining in the top layer and the right electrode (anode) in the bottom layer. Consequently, the field lines ran across the layers after the voltage was applied. After fiber-electrophoresis with the 1 kb ladder as a DNA fragment mix, elution from the corresponding segments, and visualization on an agarose gel, it can be seen that fragments of all sizes reached the position in front of the anode (no. 17). Furthermore, these experiments also show that there was a slight discrimination of fragment size. In the lowest layer, most fragments arrived in front of the anode, but here, the two largest fragments appeared to be missing. A concentration-dependent retention of the larger fragments (5000, 6000, 8000 and 10,000 bp) could also be seen in the first (no. 3) and second layer (nos. 9 and 10). Especially, the 6000 bp fragment was still prominent in the traces, as it was 2.3 times more concentrated in the original sample than the other fragments ranging from 5000 to 10,000 bp. These results imply that by stacking multiple layers of fiber material together, a size-dependent separation of the fragments might also be feasible. However, this aspect would need more investigations. Due to the small fiber thickness, the separation effect was likely due to the anisotropy of the fiber material, such that the migration of fragments across the fiber strip would result in more sample interaction than along the glass microfiber. The experiment demonstrated that the combination of glass fiber and electrode printing results in a simple separation platform that can be further advanced when reactions are enforced in the different layers. This may have particular advantages when detection platforms based on hybridization are incorporated in one of the layers. A read-out may be based on an optical [46] but also voltammetric [47,48,49] or impedimetric [50] detection of the binding of the moved DNA to an immobilized capture probe.

Compared to previous approaches, we present a simple arrangement with low interaction of the sample to be separated with the chip material and, consequently, short times for separation. A particular benefit of the microfluidic glass fiber chip is the larger geometry compared to, e.g., channels with a 1 mm width, as reported before. Typical ITP platforms with small channel dimensions allow only the analysis of very small volumes [31]. The fiber chip used in this work combines a small handling platform with the opportunity to process larger sample volumes.

## 4. Conclusions

Material properties and microfabrication simplicity let fiber substrates appear as a promising field of microfluidic technologies. Analytical separation based on the electrophoretic principle in a fiber-electrophoresis format represents a rapid separation method for DNA-containing samples. The promising capacity of glass microfiber materials make it an ideal candidate for the development of a glass microfiber-based electrophoresis of DNA samples. Chemically inert, pure binderless borosilicate glass microfiber with no additives show a low background noise in fluorescence detection and remain resistive to aggressive solvents, e.g., used in alkaline DNA extraction. The finely controlled porosity is suitable for consistent and reproducible results in microfluidic testing. The glass microfiber-electrophoresis chip described in this work provides a simple, small, and fast method to move and separate DNA from other compounds of biological samples and, thus, represents an attractive addition to former large and elaborate measurement setups used for analysis. Compared to the electrophoresis system in microfluidic chips based on silicon or plastics, the presented system is much more cost-effective. It can be used to isolate DNA from synthetic or native proteins, as well as cell debris after lysis of biological samples such as bacteria. The nucleic acid is still intact after electrophoresis and can be processed in further downstream experiments, for example, PCR or real-time PCR. Targeting of DNA samples through several layers offers the advantage of generating a size-dependent purification and to perform diverse assays in separate layer compartments in the future. With the presented development, a basis has been created for combinations with alternative detection methods, e.g., based on hybridization. Thus, chip-based separation can be coupled to in situ DNA detection. This is not only interesting from an academic point of view, but applications such as the monitoring of *Legionella* species in drinking water systems demand such robust and fast analysis platforms.

## 5. Patents

This work has been applied for a patent WO2021160641.

## Data Availability

The data presented in this study are available on request from the corresponding author.

## Supplementary Materials

The following supporting information can be downloaded at: https://www.mdpi.com/article/10.3390/bios12020062/s1, Figure S1: Image of the transfer press. Figure S2: Digital microscopic image showing a section of the chip geometry before and after the transfer to the glass microfiber. Figure S3: Measurement setup of a prototype for future read-out. Figure S4: Microscopic determination of the layer thickness of a carbon paste electrode. Figure S5: Post-staining of single fragment by intercalating dye GelRed after completing fiber electrophoresis. Figure S6: Duplicate measurement of DNA samples eluted after fiber-electrophoresis and used as a template for PCR and real-time PCR. Figure S7: Signals of the positive control with 100 ng 910 bp fragment before fiber-electrophoresis (blue) and a negative control with no template after real-time PCR with SyGreen (orange). Figure S8: HRP detection via TMB addition and verification of migrated DNA through the fiber by staining with GelRed and elution from the same chip. Figure S9: Detection of remaining proteins after fiber-electrophoresis of a *Legionella* cell lysate. Video S1: Wetting of the glass microfiber chip. Video S2. TMB oxidation by HRP after fiber electrophoresis.
