# Supplementary material for: Electrophoretic µPAD for Purification and Analysis of DNA Samples
Source: Biosensors (Basel). 2022 Jan 24;12(2):62. doi: 10.3390/bios12020062 (PMC8869226; doi:10.3390/bios12020062)
Supplement: Supplementary file 1 [file biosensors-12-00062-s001.zip › biosensors-1525168-for pub-sm.pdf]

biosensors

MDPI

Supplementary Materials

Electrophoretic μPAD for Purification and Analysis of DNA Samples

Natascha Katharina Heinsohn [1,2,*], Robert Raimund Niedl [1], Alexander Anielski [1], Fred Lisdat [3,*] and Carsten Beta [2,*]

[1] inventicsDx GmbH, Magnusstrasse 11, 12489 Berlin, Germany; r.niedl@inventicsdx.com (R.R.N.); a.anielski@inventicsdx.com (A.A.)
[2] Biological Physics, Institute of Physics and Astronomy, University of Potsdam, Karl-Liebknecht-Strasse 24/25, 14476 Potsdam, Germany;
[3] Biosystems Technology, Institute of Life Sciences and Biomedical Technologies, Technical University of Applied Sciences, Hochschulring 1, 15745 Wildau, Germany
* Correspondence: n.heinsohn@inventicsdx.com (N.K.H.); flisdat@th-wildau.de (F.L.); beta@uni-potsdam.de (C.B.)

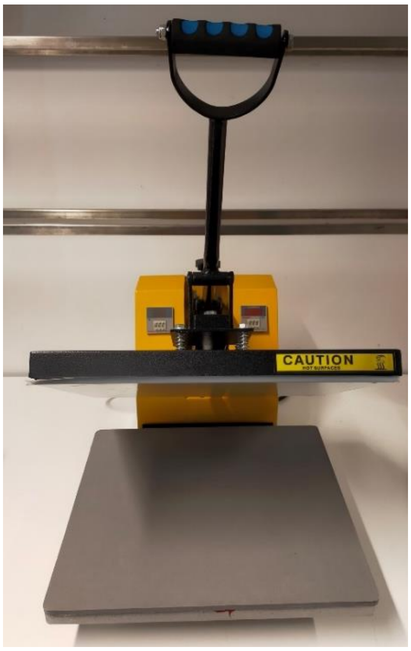

Figure S1. Image of the transfer press used for soaking of the wax barrier into the glass microfiber to define the microfluidic structure.

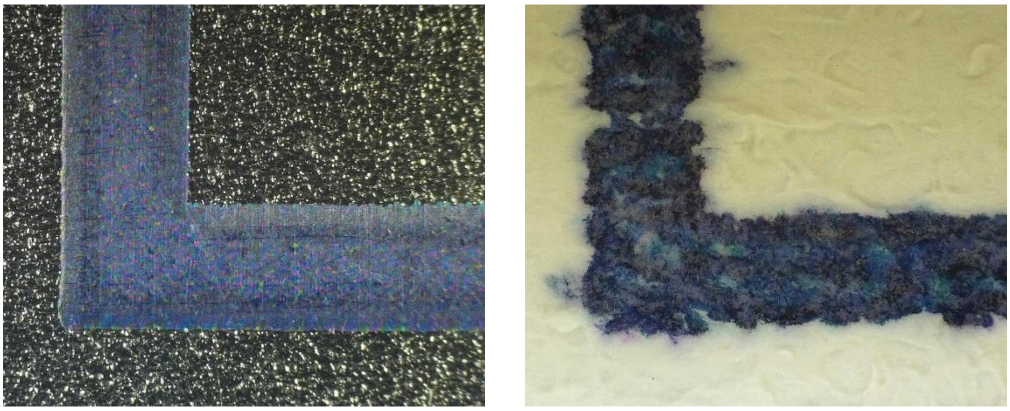

(**a**) (**b**)

Figure S2: Digital microscopic image showing a section of the chip geometry before and after the transfer to the glass microfiber. (**a**) The blue wax printed on the overhead film before transfer and (**b**) after the transfer to the glass microfiber.

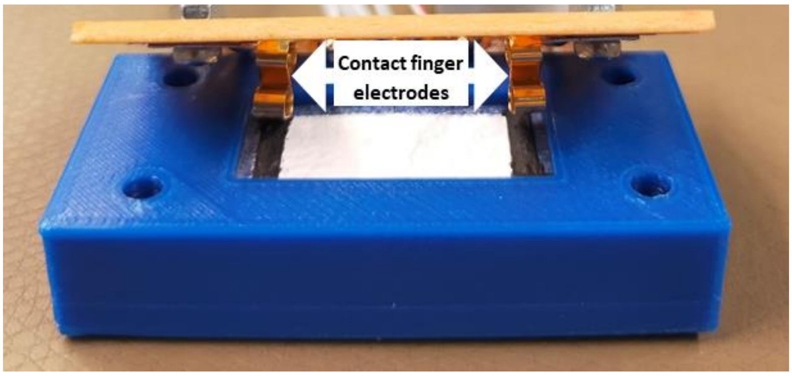

Figure S3. Measurement setup of a prototype for future read-out designed to be closer to the product (setup 2). An external electric field is applied to the glass microfiber chip through gold-coated contact fingers contacting the imprinted electrode surfaces from above.

Video S1. Wetting of the glass microfiber chip.

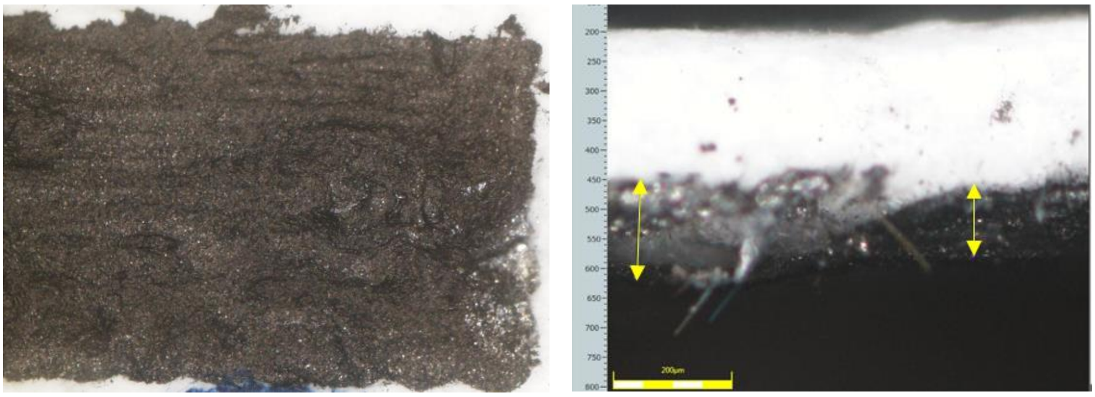

(**a**) (**b**)

Figure S4. Microscopic determination of the layer thickness of a carbon paste electrode. (**a**) Top view of the measured electrode. (**b**) Cross-section of an electrode showing here a layer thickness of 111 to 153 µm recorded with the Olympus DSX 500 optical microscope.

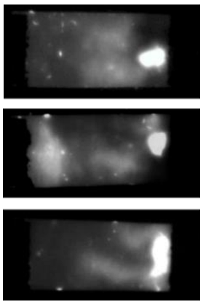

Figure S5. Post-staining of single DNA fragment by intercalating dye GelRed after completed fiber electrophoresis. Triplicates of chips with sample migrated to the end of the chip and concentrated in front of the anode.

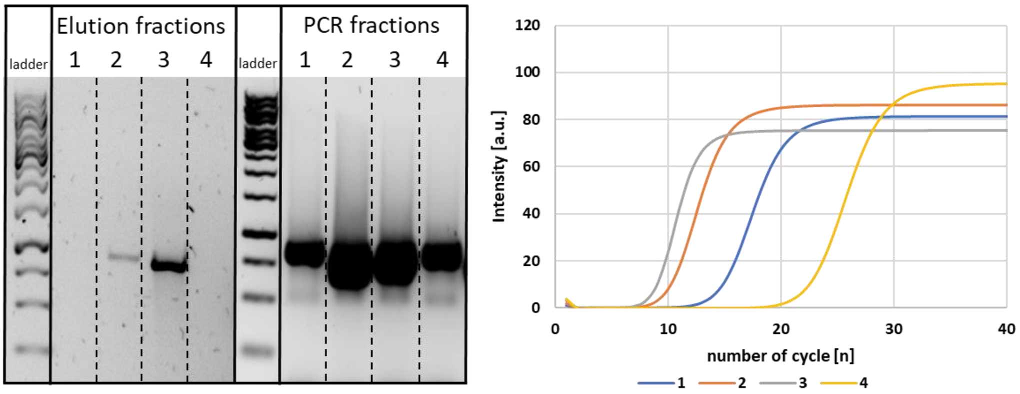

(**a**) (**b**)

Figure S6. Duplicate measurement of DNA samples eluted after fiber-electrophoresis and used as template for PCR and real-time PCR.

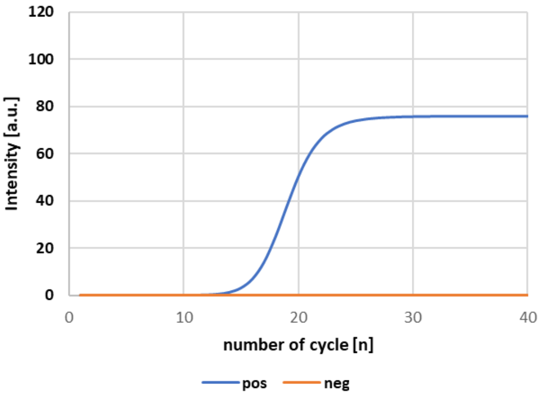

Figure S7. Signals of the positive control with 100 ng 910 bp fragment before fiber electrophoresis (blue) and a negative control with no template after real-time PCR with SyGreen (orange).

Video S2. TMB oxidation by HRP after fiber electrophoresis.

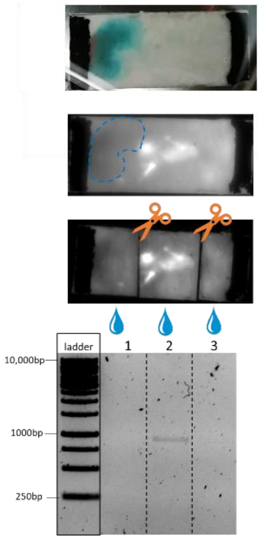

Figure S8. The immobile HRP was detected using TMB addition. DNA pulled through the fiber substrate can be both stained with GelRed on the same chip as HRP was detected and eluted if desired.

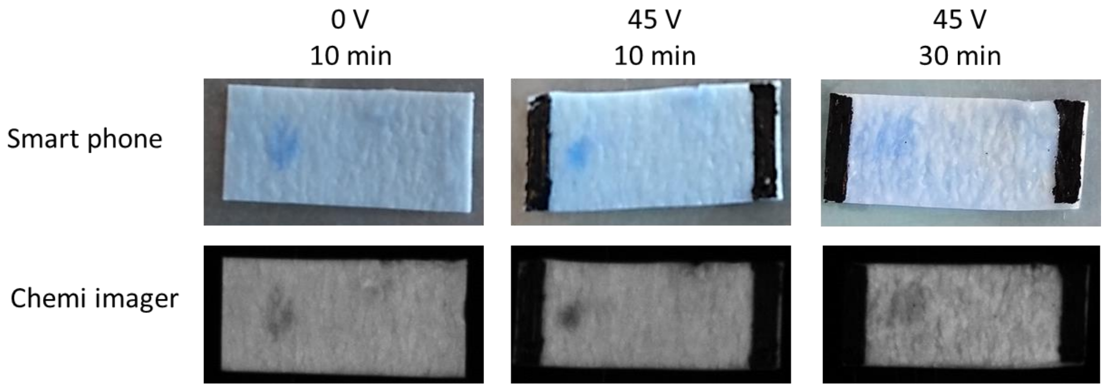

Figure S9. Detection of remaining proteins from cell lysate after fiber electrophoresis. After staining with One-Step Blue protein stain, blue spots occurred at the sample loading zone. Blue spot did not change position after 10 and 30 min indicating that proteins did migrate towards the anode.
